# The Effect of Patient Arrival Time on Overall Wait Time and Utilization of Physician and Examination Room Resources in the Outpatient Urology Clinic

**DOI:** 10.1155/2008/507436

**Published:** 2008-12-15

**Authors:** Onisuru T. Okotie, Neel Patel, Chris M. Gonzalez

**Affiliations:** Department of Urology, Northwestern University Feinberg School of Medicine, Chicago, IL 60611, USA

## Abstract

*Introduction and objective*. We examined patient waiting times, physician utilization, and exam room utilization in order to identify process improvements that may improve patient satisfaction. *Methods*. Time patient arrived to clinic, time patient was placed in the exam room, time the physician arrived in the exam room, and time physician discharged the patient from the exam room were prospectively recorded for 226 outpatient visits. *Results*. Overall, 63.2% of patients were on time for their scheduled appointment with 14.8% patient “no-shows.” On-time patients were found to have a longer wait time once in the exam room for the physician than those that were late (14.8 ± 9.2 minutes versus 11.0 ± 8.4 minutes, *P* = .005); however, those patients spent a significantly longer time with the physician (10.7 ± 6.0 minutes versus 8.9 ± 5.8 minutes, *P* = .041). Exam room utilization was lower for late patients (28.9% versus 44.7%, *P* = .03) with physician utilization lower in clinics with 3 or more late patients when compared to clinics with 2 or fewer (59.7% versus 68.7%, *P* = .004). *Conclusions*. Late patients had significantly less time with the physician than on-time patients. Late patients also decreased the overall efficiency of the clinic; therefore, measures to reduce late patients are vital to improve clinic efficiency.

## 1. INTRODUCTION

In 1999, the Institute of Medicine's (IOM) article “To err is human” reported that approximately 98000 people die annually in the US because of preventable medical mistakes [1]. This report raised public awareness of medical errors and sparked new solutions to increase health care quality. In 2001, the IOM's subsequent report “Crossing the quality chasm” recommended aligning reimbursement policies with quality improvement, increasing awareness of pay-for-performance (P4P). P4P is based on rewarding physicians for the quality of provided care with financial or other incentives [2]. Traditionally, physician reimbursement has reflected the type and quantity of care without any measurement of health care quality or patient satisfaction [3].

One important component of measured health care quality is patient satisfaction. The goal of this study was to define and analyze the impact that patient arrival time to the clinic had on overall patient flow through the office during the course of an ambulatory visit. Focus was placed on such parameters as patient waiting time, time spent with the physician, and time spent in the examination room.

## 2. MATERIALS AND METHODS

A systematic analysis of the Northwestern urology outpatient clinic process was prospectively observed over the course of three months. Parameters including patient clinic arrival time, time the patient was placed in the exam room, time the physician arrived in the exam room, and time the physician discharged the patient from the exam room. All data were recorded for 226 outpatient visits over a total of 306 scheduled appointments for one urologist cystometrogram (CMG) (Table 1). A patient was considered “late” if he/she arrived to the office check in counter after the scheduled appointment time. A patient was classified as a “no show” if he/she did not arrive for their appointment and did not call to reschedule. Exam room utilization was calculated as the ratio of total MD time in the exam room to the total patient time in the exam room. Physician utilization per clinic session was calculated as the ratio of total time the physician was in the exam room with a patient to the total time of the clinic session. An independent *t*-test was used to compare exam room and physician utilization per clinic stratified by on-time, late, new, and return patients. Patients undergoing procedure-related visits were not included in this analysis. An independent *t*-test was used to compare exam room and physician utilization per clinic stratified by on-time, late, new, and return patient status.

## 3. RESULTS

Patient flow through the outpatient urology clinic is illustrated. There were a total of 306 schedule patients included in the study analysis. Of these, 45 patients were no show patients and 35 patients were patients who underwent an office procedure. Of the 306 total scheduled patients included in the study, 63.2% were on-time or arrived before their scheduled appointment, 15.1% were <15 minutes late, and 6.8% were >15 minutes late. “No show” patients were 14.8% of the appointments (Table 2). On-time patients were found to have a statistically longer wait time once in the exam room for the physician than those who were late (14.8 ± 9.2 minutes versus 11.0 ± 8.4 minutes, *P* = .005); however on-time patients spent a significantly longer time with the physician in the exam room (10.7 ± 6.0 minutes versus 8.9 ± 5.8 minutes, *P* = .041) (Table 3).

The overall exam room utilization ratio was 32.5% and did not significantly differ between on-time and late patients (*P* = .067) or new and return patients (*P* = .35) (Table 4). When new patients were stratified as late or on-time, exam room utilization was significantly lower for late patients (28.9% versus 44.7%, *P* = .03). Furthermore, physician utilization was significantly lower in clinics with greater than 2 late patients when compared to clinics with 2 or fewer late patients (59.7% versus 68.7%, *P* = 0.004) (Table 5).

## 4. DISCUSSION

With the implementation of quality reporting, there is an increased emphasis on efficiency and patient satisfaction. We attempted to evaluate one phase of the ambulatory patient flow process, patient arrival, and analyze its impact on physician and examination room utilization. In our study, we have shown that patients who were late for their clinic appointment can be a key problem to the efficiency of the outpatient urology clinic. As the number of late patients in a clinic increases, the utilization of the physician's time significantly decreases. Furthermore, late patients were found to have spent less time with the physician, thus putting more pressure on the physician to compact the visit in a shorter amount of time in order to stay on time and maximize patient satisfaction.

Another measure of process efficiency that we analyzed was exam room utilization, defined as the ratio of the total time the physician was in the exam room to the total time the patient was in the exam room. Overall exam room utilization for all patients was poor with a mean of 32.5% use of existing capacity. Our clinic process analysis indicates that this is likely secondary to such reasons as poor communication between the office support staff and the physician as to when the patient arrives in the room and also the fact that appointments are scheduled for inappropriate amounts of time, either too long or short, where multiple patients are “overbooked” at the same time creating a scheduling bottleneck. The visit durations allowed in our electronic medical record (EMR) are 15 and 30 minutes. With the variety of patients visits categorized simply as either new or return, this inflexibility in scheduling does not capture the true amount of time needed for each visit. Many times, return postoperative patients require only 5-minute visit, whereas patients who require cancer treatment counseling may require upwards of one hour. The ability to project estimated visit times prior to the clinic session and schedule the number of patients appropriately would more closely match the demand and supply of outpatient visits and improve patient flow. Finally, we found that the need to automate patient arrival would be very helpful as many times patients arrived on time and were not properly logged into the EMR as a result of human error. In response to these data, we now ask new ambulatory clinic patients to arrive 20 minutes early and return patients 10 minutes early. Those who arrive late without warning are offered the options of waiting for the next available appointment time that day or rescheduling for another day. Data are forthcoming on the success of these strategies.

Another relevant process flaw that we have derived from these data includes the lack of stored or “prework” prior to the patient visit. New patients in our system are asked to fill out an intake questionnaire that provides basic medical information as well as insurance information. This form is typically sent to the patients before their visit as it can take up to 20 minutes to complete. Despite sending it prior to the visit, we found that only 50% of patients completed this information prior to the visit which, although they were technically on time, made them late arrivals within the confines of the process as they completed the form in the registration area. If these patients were counted as being late, the aforementioned utilization problems of this study would augmented further. Measures to improve previsit compliance with this process are needed.

Limitations of this study include the lack of postvisit patient satisfaction questionnaires which could be stratified according to the arrival time of the patient. These data would corroborate if spending more time with the physician did indeed lead to improved patients satisfaction. The use of only one physician in one office location in order to define this process may have led to specific biases commensurate with the practice patterns of that individual or institution. Multiple sites with several physicians would eliminate many of these biases. Nonetheless, these data attempt to demonstrate the complexity of patient flow processes and the ramifications of one variable, patient arrival time, within an ambulatory setting.

## 5. CONCLUSION

Late patients spent significantly less time with their physician in the exam room as compared to those who arrive early or on time. On-time or early patients spent more time with the physician, but spent longer times waiting idle in the exam room. Physician utilization per clinic session was significantly decreased in clinics with 3 or more late patients. Future improvement in the clinic process and methods to reduce the number of late arriving patients may improve the efficiency of the ambulatory patient flow process in the urology clinic.

## Figures and Tables

**Table 1:** Demographic characteristic of outpatient urology clinic patient population.

Total outpatient appointments	306
Total outpatient nonprocedure patients	226
Number of clinics	19
Mean no. of patients per clinic	15.9 ± 5.0
Mean no. of new patients clinic	4.1 ± 1.7
Mean no. of return patients per clinic	7.7 ± 3.3
Mean time in clinic per patient (minute)	34.1 ± 16.2
Mean time with MD per patient (minute)	9.0 ± 6.0

**Table 2:** Patients stratified by arrival time compared to scheduled appointment time.

Percentage of no show patients	14.8
Percentage of on time patients	63.2
Percentage of patients <15 minute late	15.1
Percentage of patients 15–30 minutes late	4.9
Percentage of patients 30–60 minutes late	1.6
Percentage of patients >60 minutes late	0.3

**Table 3:** Patient wait times by on time versus late arrival patients.

	On-time	Late	*P*-value**
Total patients (%)*	63.2	22.0	—
Mean time in exam room waiting for MD (minute)	14.8 ± 9.2	11.0 ± 8.4	**.005**
Mean time in room with MD (minute)	10.7 ± 6.0	8.9 ± 5.8	**.04**

* No show patients were 14.8%.

** *P*-values determined by independent *t*-test.

**Table 4:** Exam room utilization (*P*-values determined by independent *t*-test).

	*N*	Total	On time	Late	*P*-value*
All patients	306	0.33	0.31	0.37	.07
New patients	79	0.31	0.29	0.45	**.03**
Return patients	147	0.33	0.32	0.35	.52

* *P*-values represent difference between on-time and late groups.

**Table 5:** Physician utilization stratified by number of late patients per clinic.

	*N*	Physician	*P*-value*
		utilization	
≤ 2 late patients	9	0.69	**.02**
3 late patients	5	0.58	
≥ 4 late patients	5	0.61	

* *P*-value determined by ANOVA.
